# Thioredoxin Is Involved in Endothelial Cell Extracellular Transglutaminase 2 Activation Mediated by Celiac Disease Patient IgA

**DOI:** 10.1371/journal.pone.0077277

**Published:** 2013-10-09

**Authors:** Cristina Antonella Nadalutti, Ilma Rita Korponay-Szabo, Katri Kaukinen, Zhuo Wang, Martin Griffin, Markku Mäki, Katri Lindfors

**Affiliations:** 1 Tampere Center for Child Health Research, University of Tampere and Tampere University Hospital, Tampere, Finland; 2 Celiac Disease Center, Heim Palm Children’s Hospital, Budapest and Department of Pediatrics, Medical and Health Science Center, University of Debrecen, Debrecen, Hungary; 3 School of Medicine, University of Tampere, Department of GastroenterologyandAlimentary Tract Surgery, Tampere University Hospital, Tampere, Finland; Department of Medicine, Seinäjoki Central Hospital, Finland; 4 School of Life and Health Sciences, Aston University, Birmingham, United Kingdom; Institute for Virus Research, Laboratory of Infection and Prevention, Japan

## Abstract

**Purpose:**

To investigate the role of thioredoxin (TRX), a novel regulator of extracellular transglutaminase 2 (TG2), in celiac patients IgA (CD IgA) mediated TG2 enzymatic activation.

**Methods:**

TG2 enzymatic activity was evaluated in endothelial cells (HUVECs) under different experimental conditions by ELISA and Western blotting. Extracellular TG2 expression was studied by ELISA and immunofluorescence. TRX was analysed by Western blotting and ELISA. Serum immunoglobulins class A from healthy subjects (H IgA) were used as controls. Extracellular TG2 enzymatic activity was inhibited by R281. PX12, a TRX inhibitor, was also employed in the present study.

**Results:**

We have found that in HUVECs CD IgA is able to induce the activation of extracellular TG2 in a dose-dependent manner. Particularly, we noted that the extracellular modulation of TG2 activity mediated by CD IgA occurred only under reducing conditions, also needed to maintain antibody binding. Furthermore, CD IgA-treated HUVECs were characterized by a slightly augmented TG2 surface expression which was independent from extracellular TG2 activation. We also observed that HUVECs cultured in the presence of CD IgA evinced decreased TRX surface expression, coupled with increased secretion of the protein into the culture medium. Intriguingly, inhibition of TRX after CD IgA treatment was able to overcome most of the CD IgA-mediated effects including the TG2 extracellular transamidase activity.

**Conclusions:**

Altogether our findings suggest that in endothelial cells CD IgA mediate the constitutive activation of extracellular TG2 by a mechanism involving the redox sensor protein TRX.

## Introduction

Transglutaminase 2 (TG2) is a ubiquitously expressed highly complex multifunctional protein with enzymatic, cell adhesion, cell signaling and G-protein activities [1,2]. These functions of TG2 are regulated by other proteins and cofactors, including Ca^2+^ and GTP, and by the availability of these modulators in different tissue compartments [3,4]. In the extracellular environment, the abundance of Ca^2+^ and the low GTP/GDP nucleotide concentrations may allow TG2 to act as an enzyme modifying proteins at post-translational level by transamidation or deamidation [5] while this activity is probably repressed in the cytoplasm, where TG2 can act as a GTP-ase. Cell surface TG2 is implicated in cell adhesion by protein-protein interactions. As a transamidase, TG2 catalyzes the formation of isopeptide bonds (ε-γ-glutamyl- lysine crosslink) between a selected glutamine residue on one substrate and a lysine residue on another. In the absence of adequate concentrations of any suitable amine substrate, TG2 deamidates the targeted glutamine side chain, resulting in its conversion to glutamate [6].

The enzymatic activity of TG2 is of research interest in that it is implicated in the pathogenesis of a number of human conditions, including celiac disease, a dietary gluten-induced autoimmune-mediated enteropathy affecting HLA-DQ2 or HLA-DQ8 subjects [7]. In celiac disease, TG2 enzymatic activity is known to lead to the deamidation of gluten peptides, which increases their affinity for HLA-DQ2 or HLA-DQ8 molecules [8]. This triggers an inflammatory T cell response ultimately resulting in the destruction of the small-bowel mucosal architecture [9]. Furthermore, during the disease pathogenesis the patients mount an anti-TG2 autoantibody response [10]. These autoantibodies are present in the serum and in various tissues in the patients, particularly in the small-intestinal mucosa as deposits on the subepithelial basement membrane and around capillary walls [11,12]. Intriguingly, the small-bowel mucosal TG2 located at the sites where the antibodies bind has been suggested to be catalytically active in untreated celiac patients [13]. It has thus been proposed that celiac disease-specific autoantibodies might account for the enzymatic activation of small-bowel mucosal TG2, although contrasting results are also reported [14].

Our previous studies have suggested that celiac IgA increases TG2 enzymatic activity on endothelial cells, but the mechanism involved has not yet been characterized [15,16]. Interestingly, it has recently been shown that thioredoxin (TRX), a redox sensor protein in cells, is a novel regulator of extracellular TG2 activity [17]. Therefore, the present aim was thus to deepen the understanding of CD IgA-mediated TG2 enzymatic activation and to establish whether TRX might be a key molecule in this process.

## Materials and Methods

### Cell Culture and Reagents

Human umbilical vein endothelial cells (HUVECs) were purchased from Lonza (Cambrex Bio Science, Walkersville, MD) and maintained in EGM-2 medium (Clonetics®, San Diego, CA) with 2% fetal bovine serum (FBS) (Gibco Invitrogen, Paisley, Scotland, UK) and 25 µg/mL endothelial cell growth supplements (Clonetics). In all the experiments performed, the cells were used between passages 2 and 6. A non-reversible and cell-impermeable inhibitor for extracellular TG2 activity, R281 [18], was used throughout at a final concentration of 200 µM. In our experimental settings we employed also the TRX inhibitor PX-12 (1-methylpropyl 2-imidazolyl disulfide) at a non-toxic concentration of 1 µM (TOCRIS, Bioscience, Ellisville, MI). All the chemical compounds were administered 1h before addition of serum IgA.

### Purification of serum IgA class antibodies

Serum samples from nine biopsy-proven celiac disease patients on a gluten-containing diet and five non-celiac healthy controls were included in the present study. All celiac sera were positive for anti-TG2 antibodies (titer ≥100 U/mL) (Celikey®; Pharmacia Diagnostic GmbH, Freiburg, Germany) and endomysial antibodies (titer 1≥1000), while all control sera were negative. Total serum IgA from healthy donors (H IgA) and celiac patient sera (CD IgA) were purified as previously described [11] and diluted in PBS to a final concentration of 100 µg/mL. Purified IgA fractions, if not otherwise stated, were used in all experiments at a concentration of 1 µg/ml.

### Ethics Statement

The study protocol was approved by the Ethics Committee of Tampere University Hospital, Tampere, Finland, and all patients gave written informed consent.

### TG2 activity assays

In order to determine the extracellular transamidating TG2 activity HUVECs were plated onto commercial fibronectin (FN)-pre-coated 96-well plates (BD Biosciences Discovery Labware, Bedford, MA) at a density of 2x10^5^ cells per well and grown to confluence. Thereafter, the cells were starved overnight (O/N) in EGM-2 containing 1% of FBS followed by incubation in the presence of different study compounds. At indicated time-points the cells were washed with Hanks Balanced Salt Solution (HBSS; Sigma Aldrich, St. Louis, MI), followed by incubation with the TG2 substrate monodansylcadaverine (MDC) (0.2 mM; Sigma Aldrich) for 2h at 37°C. Subsequently, the wells were extensively washed and the layers fixed with 4% paraformaldehyde (PFA) (Sigma Aldrich). TG2-mediated MDC incorporation was detected using rabbit anti-dansyl antibody for 30 min at 37°C (1:1000; Invitrogen, Carlsbad, CA). A secondary antibody, horseradish peroxidase-conjugated polyclonal swine anti-rabbit immunoglobulin (1:2000; Dako, Copenhagen, Denmark), was then added to the plates and incubated for 30 min at 37°C. Finally, the peroxidase substrate 3,3’,5,5’-tetramethylbenzidine (TMB) (Slow Kinetic Form for ELISA; Sigma Aldrich) was used and the enzymatic reaction stopped with 2.5 M H_2_SO_4_. Absorbance was measured at 450 nm by spectrophotometer (Multiskan Ascent; Thermo Labsystems, Waltham, MA). In order to demonstrate TG2-specific incorporation of MDC, R281, was used.

Due to the fact that TG2 is ubiquitously expressed we further investigated where the enzyme was activated, focusing on the extracellular matrix environment. To this end, HUVECs grown to confluence as described above, were dislodged from their substratum by cell dissociation buffer (PBS, pH 7.4, with 2 mm EDTA), leaving the cell-assembled matrix (ECM). Thereafter, the plates were examined as described.

For Western blotting analyses of extracellular TG2 activity, the ECM fractions, obtained upon EDTA treatment, were directly solubilized by the addition of Laemmli gel loading buffer. The samples were loaded and run on 12% SDS-PAGE gels using the Bio-Rad Mini-Protein Tetra Cell system (Bio-Rad Laboratories, Espoo, Finland). Proteins were transferred to nitrocellulose filters (Hybond C-extra; Amersham Biosciences, Little Chalfont, UK) and non-specific binding was blocked with 5% non-fat powdered milk for 1h at room temperature (RT). The membranes were then incubated overnight (O/N) at +4°C with rabbit anti-dansyl antibody (1:10000; Invitrogen). Thereafter, immunoreactivity was detected by consecutive incubation of the membranes with polyclonal swine anti-rabbit horseradish peroxidase (HRP)-conjugated secondary antibody (Dako, Glostrup, Denmark) for 1h at RT. The bands were visualized using the chemiluminescence detection system (Amersham, ECL Plus Western Blotting Detection System, Buckinghamshire, UK) and quantified by the Kodak 1D Image analyses software (Kodak, New Haven, CT). As loading control, γ-tubulin (GTU-88, Sigma Aldrich) was used. As above, R281 was used to demonstrate TG2 specificity of MDC incorporation.

Additionally, to investigate the relationship between the transamidating extracellular TG2 activity and the amount of CD IgA, we have designed a dose response test based on the microtiter plate assay using live cells. Briefly, HUVECs were plated on FN pre-coated 96-well plates (BD Biosciences Discovery Labware) and cultured to confluence in EGM-2 medium. The day prior to the experiment, the cells were starved O/N in EGM-2 medium (Clonetics) containing 1% FBS (Gibco Invitrogen) and then treated with different CD IgA concentrations (0.5 µg/mL; 1 µg/mL; 5 µg/mL; 25 µg/mL) for 3 h at 37°C, 5% CO_2_. Thereafter, in situ-TG2 activity was investigated as described above except that the cells were not removed from the plates using a modification of the in situ assay by Verderio [19].

### Quantification of extracellular TG2 expression on endothelial cells

In order to investigate the extracellular surface expression of TG2 by ELISA, HUVECs were grown to confluence on commercial FN-pre-coated 96-well plates (BD Biosciences Discovery Labware). After O/N starvation in EGM-2 with 1% of FBS, the HUVECs were incubated for 24h in the presence of different study compounds. The cells were then washed with HBSS (Sigma Aldrich), fixed with 4% PFA (Sigma Aldrich) and blocked with 5% BSA (Sigma Aldrich). The plates were incubated either with mouse monoclonal antibody CUB7402 TG2 (NeoMarkers, Fremont, CA) or with an anti-TG2 antibody recognizing the FN-binding domain, (clone 4G3, Millipore, MA) for 1h at 37°C. Subsequently, the wells were extensively washed and a specific secondary HRP-conjugated anti-mouse immunoglobulins (1:2000; Dako) was added for 30 minutes at 37°C. Finally, the peroxidase substrate TMB (Sigma Aldrich) was used and the enzymatic reaction was stopped with 2.5 M H_2_SO_4_. The absorbance was measured at 450 nm by spectrophotometer (Multiskan Ascent; Thermo Labsystems).

### In vitro redox modulation of TG2 bound FN

In order to establish whether CD IgA is able to activate oxidized inactive TG2 we used a modified protocol by Stamnaes and coworkers [17]. The assay is based on the knowledge that TG2 is sensitive to oxidative environments and gradually loses activity upon handling and storage due to oxidation [20]. In this system, Human FN 96-well MultiwellTM (BD Bioscences, Bedford, Massachussets, USA) were coated with 5µg/mL human recombinant TG2 (His-rhTG2) (Zedira GmbH, Darmstadt, Germany) in Tris-buffered saline for 1h at 37°C. Enzymatic activity was measured by addition of 40 µM 5-biotinamidopentylamine (5-BP) (Life Science Research Pierce Biotechnology, Rockford, IL) under different sample conditions. In the experiments Ca^2+^ was used at the concentration of 10 mM and dithiotreitol (DTT) at 1 mM. After extensive washings, incorporated 5-BP was detected with streptavidin HRP-conjugated secondary antibody (Life Science Research Pierce Biotechnology). Lastly, the peroxidase substrate TMB (Sigma Aldrich) was used and the enzymatic reaction stopped with 2.5 M H_2_SO_4_. Absorbance was measured at 450 nm by spectrophotometer (Multiskan Ascent; Thermo Labsystems). In order to verify that TG2 remained immobilized during the procedure, some wells were incubated with CUB7402 (1:200) (NeoMarkers, Fremont, CA) instead of 5-BP.

### Binding of celiac autoantibodies to TG2 after redox modulation

TG2 purified from guinea pig liver (5 µg/mL) (Sigma Aldrich) was coated on microtiter plates (Maxisorp, Nunc A/S, Roskilde, Denmark) for 1 hour at RT in Tris-buffered saline with 5 mM CaCl_2_. After extensive washings with Tris-buffered saline with 10 mM EDTA and 0.1% Tween 20, the plates were treated with 100 µM 5,5'-dithiobis-(2-nitrobenzoic acid) (DTNB) for 20 minutes, followed by incubation with 50 mM DTT for another 20 minutes and further washings. Serum samples from six celiac patients diluted 1:200 or CUB7402 (1:16000) (NeoMarkers) were added in triplicate for 30 minutes. Bound antibodies were measured according to Sulkanen and coworkers [21].

### Thioredoxin assay

The expression and release of thioredoxin was evaluated in endothelial cells by ELISA and Western blotting. For ELISA, HUVECs were plated onto commercial FN pre-coated 96-well plates (BD Biosciences Discovery Labware) at a density of 1x10^4^ cells per well. The cells were grown in complete culture media until confluence and starved O/N before the assay. Thereafter, the HUVECs were incubated with purified IgA class antibodies from healthy donors and celiac patients for 24h at 37°C, with or without pre-incubation with different inhibitors. The cells were then washed, fixed with 4% PFA (Sigma Aldrich) and blocked in 2% BSA buffer. Surface expression of thioredoxin on endothelial cells was detected by incubation of the plates using mouse anti-thioredoxin antibody (1:200) (sc-166393, Santa Cruz Biotechnology, Scotts Valley, CA) for 1h at 37°C, followed by incubation with the specific HRP-conjugated secondary antibody (1:2000; Dako) Finally, the peroxidase substrate TMB (Sigma Aldrich) was used and the enzymatic reaction stopped with 2.5 M H_2_SO_4_. Absorbance was measured at 450 nm by spectrophotometer (Multiskan Ascent; Thermo Labsystems).

For Western blotting analyses, HUVECs were cultured in complete endothelial growth medium in 24-well plate format and starved in EGM-2 medium (Clonetics) with 1% FBS (Gibco Invitrogen) O/N before the assay. The cells were then treated for 24h with H IgA or with CD IgA, preceded by pre-incubation with specific inhibitors, as described above. Thereafter, the culture medium was collected, briefly centrifuged and solubilized by addition of Laemmli buffer. Whole cell lysates were obtained by direct solubilization and extraction of proteins from cell monolayers in Laemmli buffer. Lastly, cell culture supernatants and whole cell lysates were subjected to Western blotting, using as primary antibodies mouse anti-TRX antibody (1:2000) (Santa Cruz Biotechnology) and γ-tubulin (Sigma Aldrich) as loading control. Immunoreactivity was detected as above by sequential incubation of the membranes with appropriate HRP-conjugated secondary antibody (Dako) for 1h at RT. Visualization was by the chemiluminescence detection system (Amersham), ECL Plus Western Blotting Detection System) and quantification by the Kodak 1D Image analyses software (Kodak).

### Human INF-ƴ assay

Quantitative measurement of human INF-ƴ in culture supernatants of endothelial cells under different experimental conditions was implemented by an *in vitro* enzyme-linked immunosorbent assay according to the manufacturer’s instructions (Fisher Scientific, Waltham, MA).

### Immunofluorescent stainings

HUVEC cells were cultured on FN-precoated chamber slides (BD-Falcon™, Bedford, MA) under different experimental conditions. The cells were then fixed in 4% PFA, washed with PBS1x and blocked with 1% BSA. Immunolabelings were carried out using as primary antibody anti-TG2 (diluted 1:50) (clone 4G3, Millipore) detected by Alexa Fluor® 488-conjugated secondary anti-mouse antibody (1:3000) (Invitrogen, Molecular Probes™, Leiden, the Netherlands). Stainings were visualized by fluorescent microscopy analysis (Olympus Optical CO.Ltd BX60F5, Tokyo, Japan).

### Statistical analysis

Statistical analysis was performed using the non-parametric Mann–Whitney U-Test and the data were presented as mean values. A p-value <0.05 was considered statistically significant.

## Results

### Effects of celiac IgA on endothelial cell extracellular transglutaminase 2 transamidating activity and amount

**Figure 1:**
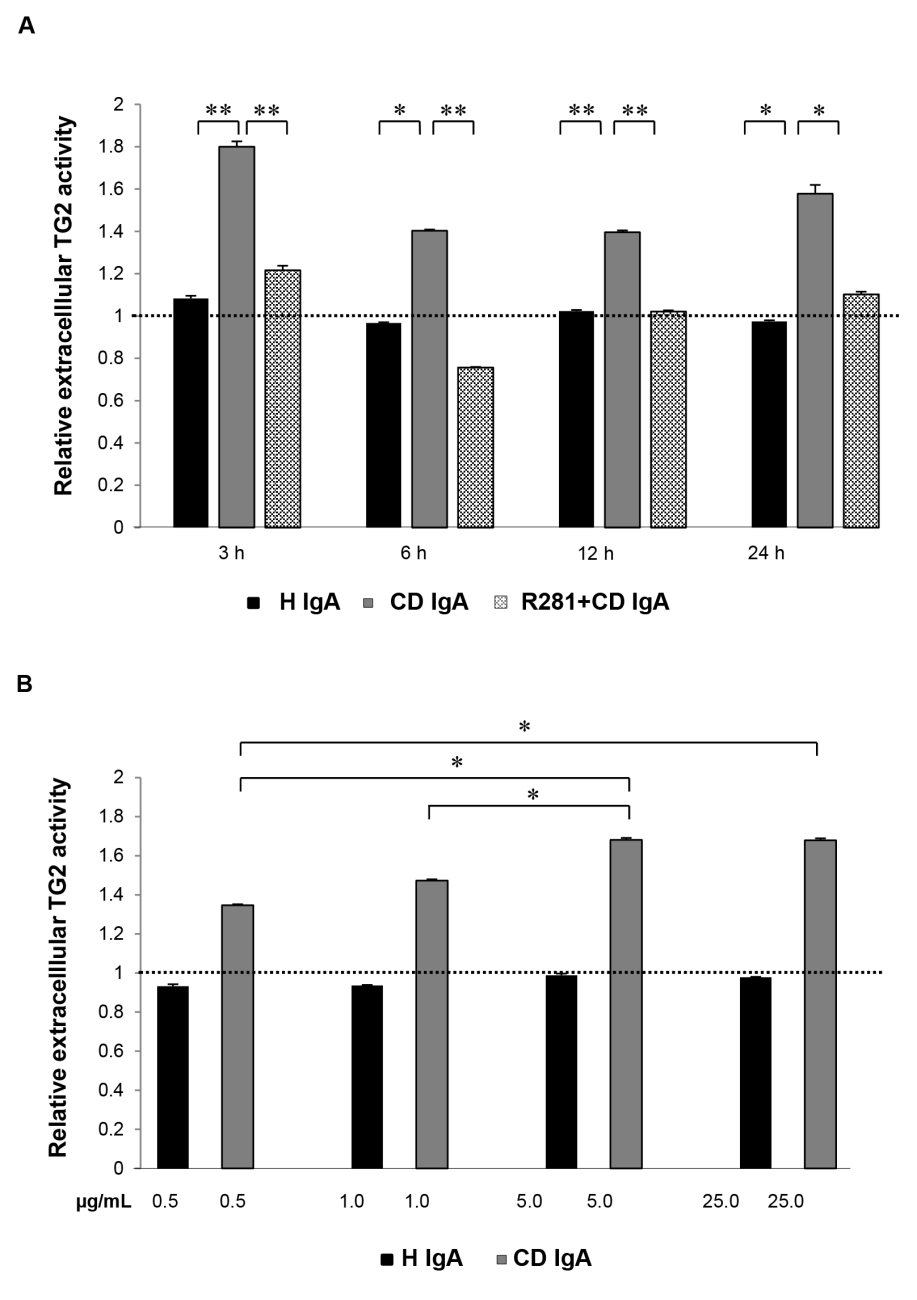
Endothelial extracellular transglutaminase 2 (TG2) activity analyses by monodansylcadaverine (MDC) incorporation. The relative extracellular TG2 activity (**A**) at different time points and with (**B**) increasing concentrations of class A immunoglobulins are shown. The dashed line indicates TG2 activity in untreated endothelial cells (HUVECs). Bars represent mean MDC incorporation as fold of control and error bars indicate standard error of the mean. P-value < 0.05 was considered significant ( _**_p<0.001 and _*_p<0.01). Data derived from three independent experiments, repeated in quadruplicate. HUVECs were treated with celiac disease patient (CD IgA) and non-celiac subject’s immunoglobulin-A (H IgA). Extracellular TG2 activity was inhibited by administering a non-permeable, site directed TG2 inhibitor, R281.

In order to establish whether CD IgA increases the endothelial cell extracellular transamidating activity of TG2 in a time- and/or dose-dependent manner we performed an *in situ* TG2 activity assay with confluent HUVECs monolayers starved O/N. We observed that extracellular TG2 activity in HUVECs was only present on addition of CD IgA but not when H IgA was used (Figure 1A). With CD IgA extracellular TG2 enzymatic activity was already detectable after 3h of treatment. Although at this time-point maximal activation of the enzyme was observed, the induction was detectable at all-time points considered. Pretreatment of the non-permeable, site-directed TG2 inhibitor R281 abolished the increased extracellular TG2 transamidating activity induced by CD IgA throughout the experiment. To study the dose-dependent relationship between CD IgA-mediated extracellular TG2 activation, we added different amounts of IgA, ranging from 0.5µg/mL to 25µg/mL, to cultured endothelial cells. Interestingly, there was a dose-dependent rise in extracellular TG2 enzymatic activity in HUVECs incubated with increasing CD IgA concentrations (Figure 1B). The maximal activity of 1.7-fold was reached with 5 µg/mL, after which no further increase was achieved. Inhibitor R281 had no effect on the enzymatic activity when administered (data not shown).

**Figure 2:**
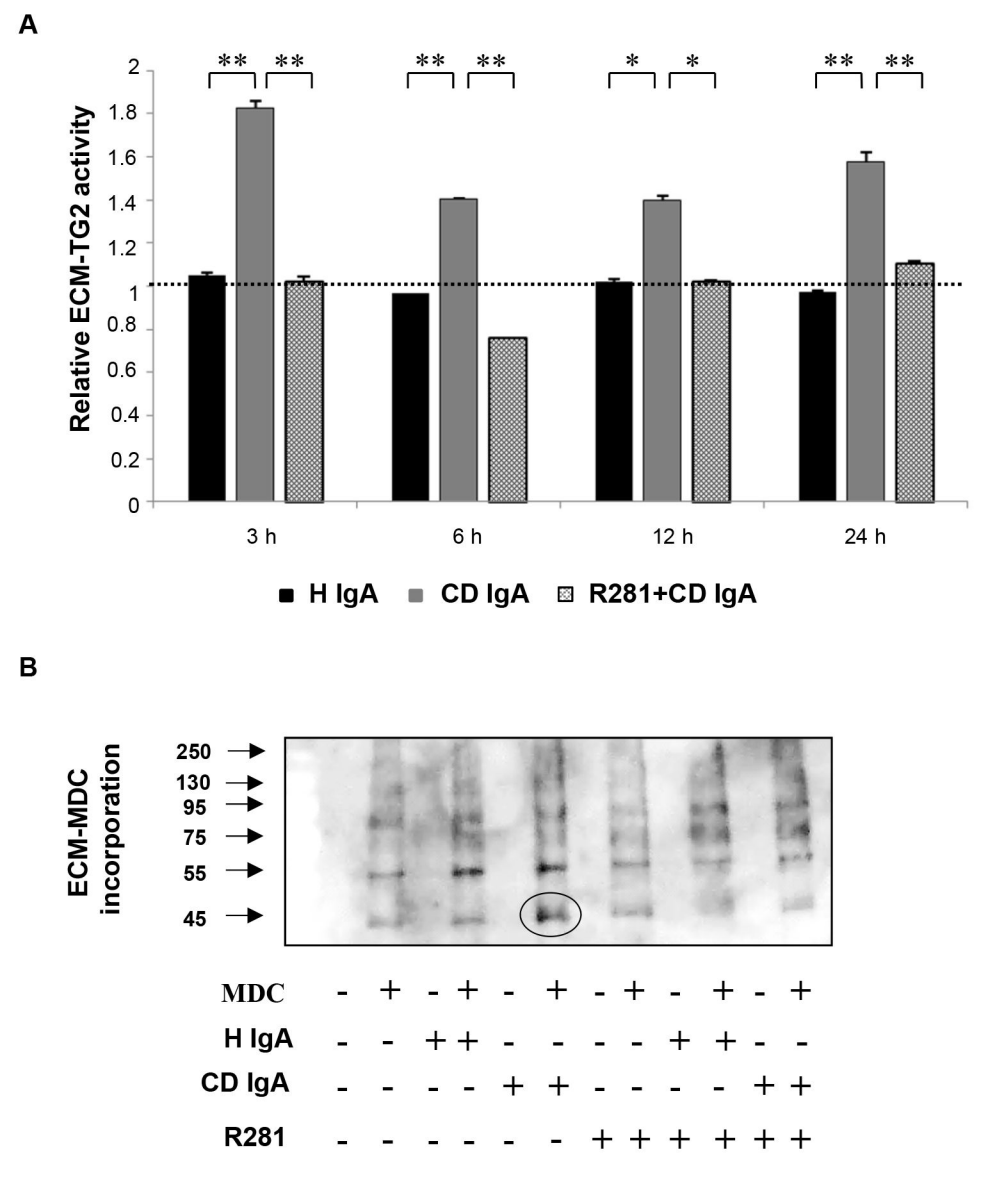
Endothelial extracellular matrix (ECM) transglutaminase 2 (TG2) activity analyses by monodansylcadaverine (MDC) incorporation. (**A**) The relative ECM-TG2 activity at different time points by ELISA. The dashed line indicates TG2 activity in untreated endothelial cells (HUVEC). Bars represent mean MDC incorporation as fold of control and error bars indicate standard error of the mean. P-value < 0.05 was considered significant ( _**_p<0.001 and _*_p<0.01). Data derived from three independent experiments, repeated in quadruplicate. (**B**) Western blotting analyses of endothelial ECM-TG2 activity measured by the incorporation of MDC. Endothelial cells were treated with celiac disease patient (CD IgA) and non-celiac subject’s immunoglobulin-A (H IgA). Extracellular TG2 activity was inhibited by administering a non-permeable, site directed TG2 inhibitor, R281.

Since endothelial cells express high levels of TG2 on the cell surface but also secrete it into their ECM, we sought further to investigate the precise localization of the TG2 activity stimulated by CD IgA. To this end, we studied TG2-mediated incorporation of MDC into the ECM fractions laid down by HUVECs, under different experimental conditions. We identified a statistically significant increase in relative ECM-TG2 activity when CD IgA was administered to cultured endothelial cells (Figure 2A). The observed effect was comparable to that detected on cell monolayers over time. Further, we collected deoxycolate-insoluble matrix protein under different experimental conditions and subjected them to Western blotting analyses in order to visualize MDC incorporation into ECM (Figure 2B). We detected several bands spread over a wide molecular weight range in all treatment groups. However, in the ECM derived from endothelial cells incubated with CD IgA there was an increase in the signal of low molecular mass proteins when compared to control groups, as indicated by the black circle in Figure 2B. In samples derived from cultures with R281 and CD IgA the intensity of the 45 kDa band was clearly reduced and similar to the controls.

**Figure 3:**
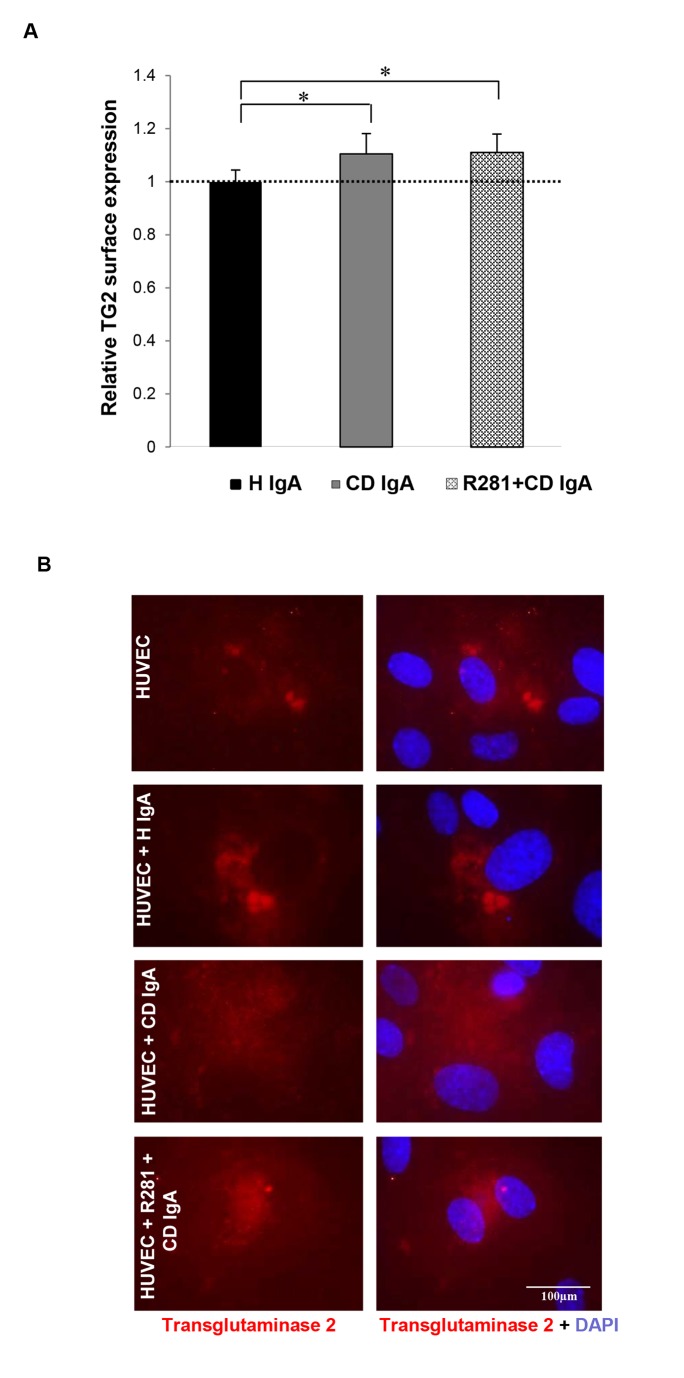
Celiac patient immunoglobulins A (CD IgA) increase transglutaminase 2 (TG2) surface deposition on endothelial cells. (**A**) The relative surface expression of TG2 on non-permeabilized endothelial cells (HUVECs) was studied by ELISA. The dashed line indicates TG2 surface expression in untreated HUVECs. Bars represent mean TG2 expression as fold of control and error bars indicate standard error of the mean. P-value <0.05 was considered significant ( _*_p<0.001). Data derived from three independent experiments, repeated in quadruplicate are shown. (**B**) Surface expression of TG2 on non-permeabilized HUVECs was also investigated by immunofluorescence (IF) with the monoclonal TG2 antibody, 4G3. Representative IF stainings are shown. Control non-celiac subject’s immunoglobulin-A (H IgA). Extracellular TG2 activity was inhibited by administering a site directed, non-permeable inhibitor, R281.

As HUVECs in the presence of CD IgA are characterized by increased extracellular TG2 transamidating activity, we sought to ascertain whether this was associated with augmented extracellular TG2 expression. The relative quantification of cell surface-associated TG2 in non-permeabilized HUVECs with 4G3 demonstrated that in the presence of CD IgA there was a 1.1-fold increase in extracellular TG2 (Figure 3A and 3B). Comparable results were achieved with CUB7402 which recognizes a linear TG2 epitope (data not shown). Co-administration of the non-reversible and cell impermeable inhibitor for extracellular TG2 activity R281 was not able to prevent this effect (Figure 3A and 3B).

### Redox regulation of TG2 is important for the enzymatic activation by CD IgA

**Figure 4:**
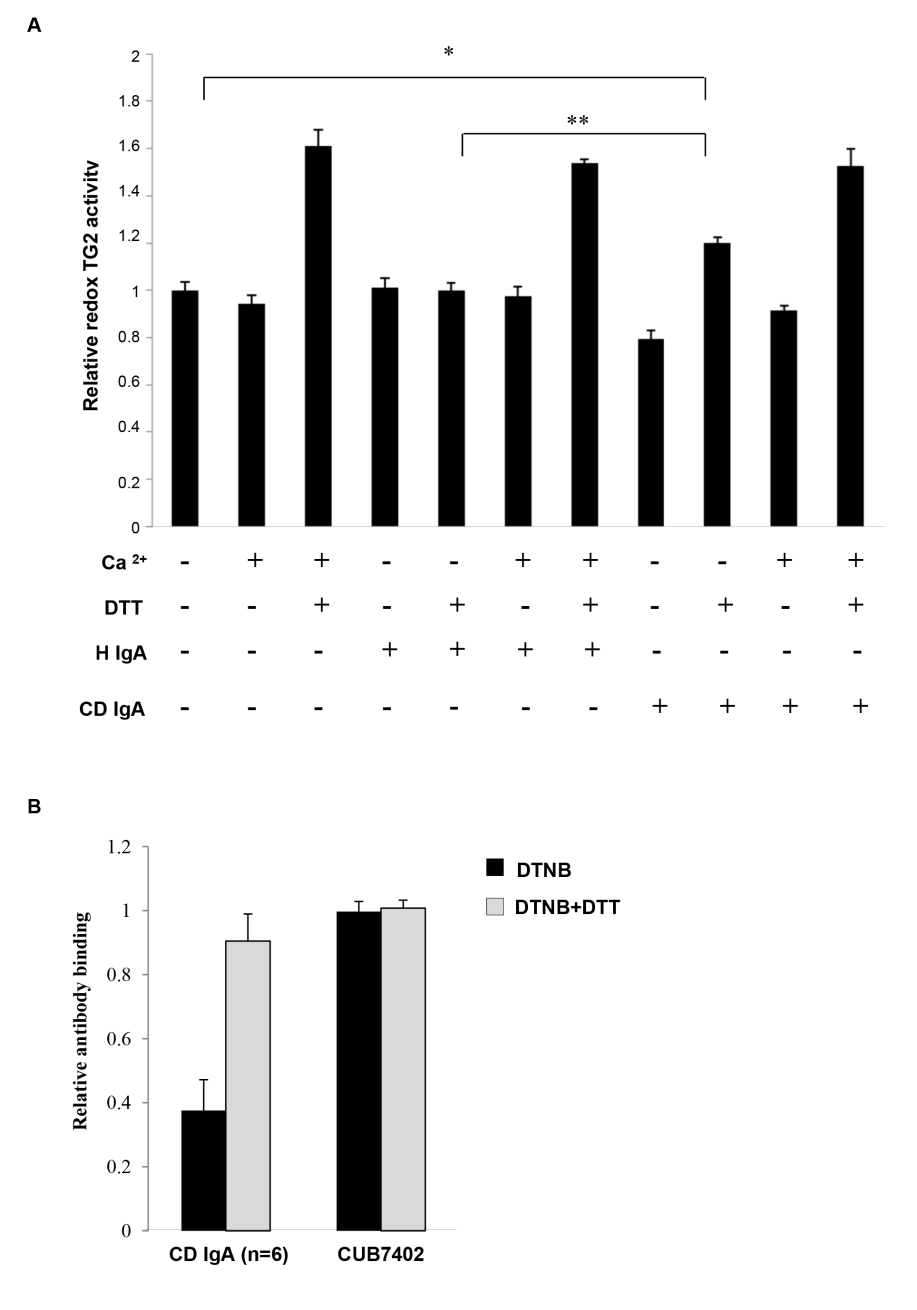
Celiac disease IgA (CD IgA) can activate fibronectin-bound transglutaminase 2 (FN-TG2) under reducing conditions. The effect of CD IgA on TG2 enzymatic activity was assessed by EZ-Link Pentylamine-Biotin (5-BP) incorporation on FN. In the absence of both the reducing agent, dithiothreitol (DTT) and Ca^2+^, TG2, alone or with any IgA, was enzymatically inactive. Maximal TG2 activity was obtained in the presence of DTT and Ca^2+^ even in the presence of class A immunoglobulins. On the contrary, in the presence of a reducing agent, DTT, but without exogenous Ca^2+^, CD IgA was able to induce the activation of TG2 in contrast to non-celiac IgA (H IgA). Bars represent mean 5-BP incorporation on FN as fold of TG2 in the absence of DTT and Ca^2+^. Error bars indicate standard error of the mean; P-value < 0.05 was considered significant ( _***_p<0.001, _**_p<0.01 and _*_p<0.05). Data derived from three independent experiments, repeated in quadruplicate are shown.

As previous studies have shown that extracellular TG2 activity is sensitive to redox changes, we next investigated whether the redox regulation of TG2 might also have an effect on the ability of CD IgA to activate the enzyme. We observed that in the absence of a reducing agent DTT and exogenous Ca^2+^, TG2 was enzymatically inactive and both agents were required for maximal activity (Figure 4A). The addition of CD IgA or H IgA did not affect the transamidating TG2 activity either in the presence or in the absence of both Ca^2+^ and DTT. Interestingly, in the presence of DTT but without exogenous Ca^2+^, CD IgA autoantibodies were able to induce enzymatic activity of TG2 in contrast to H IgA. We also observed that under oxidizing conditions induced by DTNB, reduced amounts of CD IgA bind to the enzyme and that the process can be reversed by treating the enzyme with DTT after DNTB and before adding the antibodies. Neither DTNB nor DTT had any effect on the binding of anti-TG2 monoclonal antibody CUB7402, which recognizes a linear epitope (Figure 4B).

### Celiac IgA-mediated activation of extracellular transglutaminase 2 requires thioredoxin

**Figure 5:**
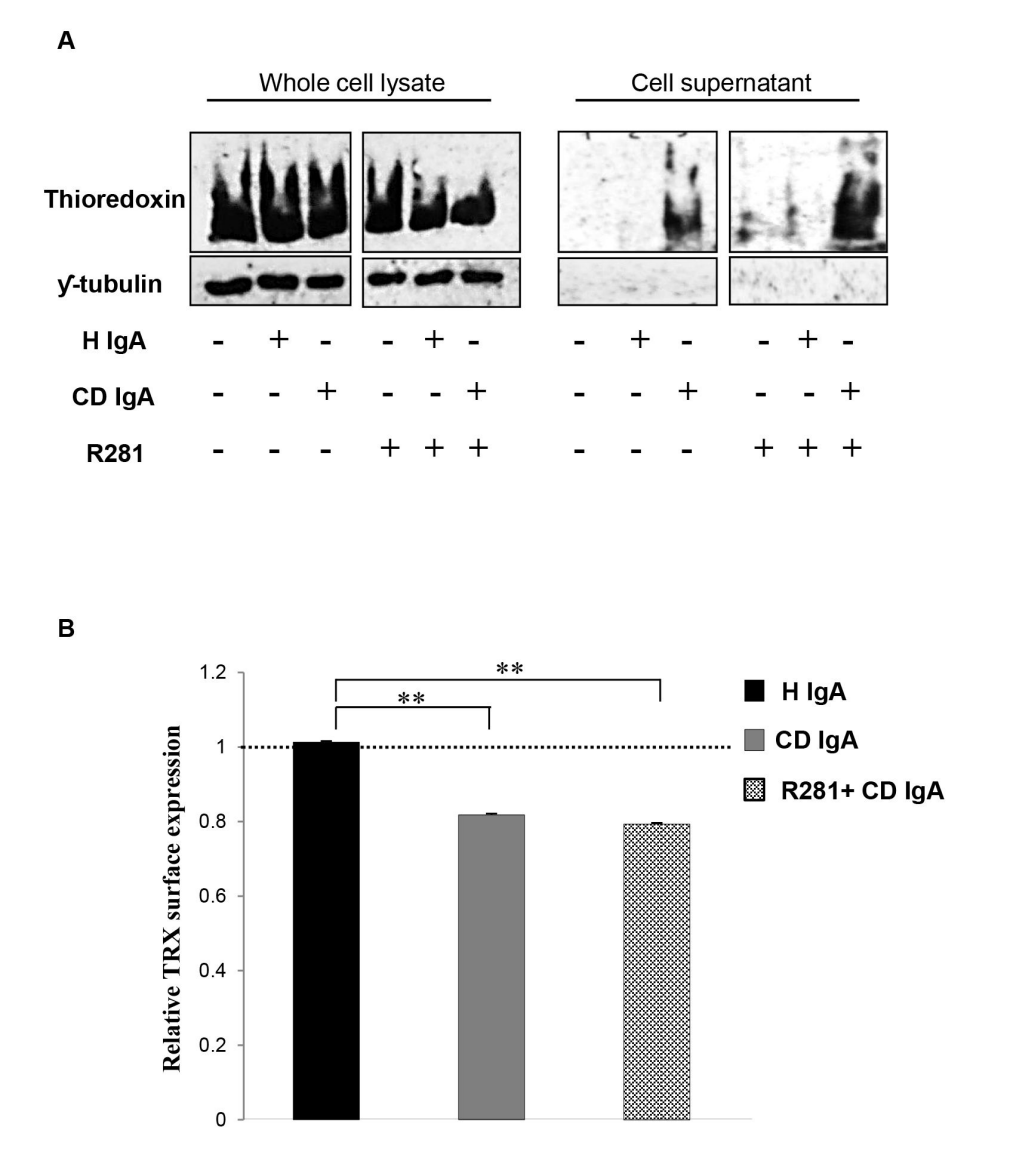
Celiac IgA (CD IgA) promotes secretion of thioredoxin. **(TRX) and decreases its endothelial surface expression**. (**A**) Representative Western blots from total cell lysates and supernatants show amounts of TRX and ƴ-tubulin used as loading control for protein extracts. (**B**) Relative surface expression of TRX was studied by ELISA. The dashed line indicates TRX surface expression in untreated HUVECs. Bars represent mean TRX expression as fold of control and error bars indicate standard error of the mean. P-value <0.05 was considered significant ( _*_p<0.05 and _**_p<0.001). Data derived from three independent experiments, repeated in quadruplicate are shown. Endothelial cells were treated with CD IgA and non-celiac subject’s immunoglobulin-A (H IgA). Extracellular TG2 activity was inhibited by administering a site directed, non-permeable inhibitor, R281.

Since we found that CD IgA mediated TG2 activation occurs only under reducing conditions we sought to establish whether the novel redox TG2 regulator TRX was involved in the process as a physiological reducing agent. We found that the overall TRX protein expression level to be comparable in all treatment groups (Figure 5A). On the other hand, endothelial cells incubated with CD IgA secreted TRX into the cell cultured media, whereas control cells did not. TRX secretion was coupled to its decreased surface expression in CD IgA-treated HUVECs (Figure 5B). It was of note that, co-administration of the inhibitor for extracellular TG2 activity, R281, was not able to prevent these effects exerted by CD IgA (Figure 5A and 5B).

**Figure 6:**
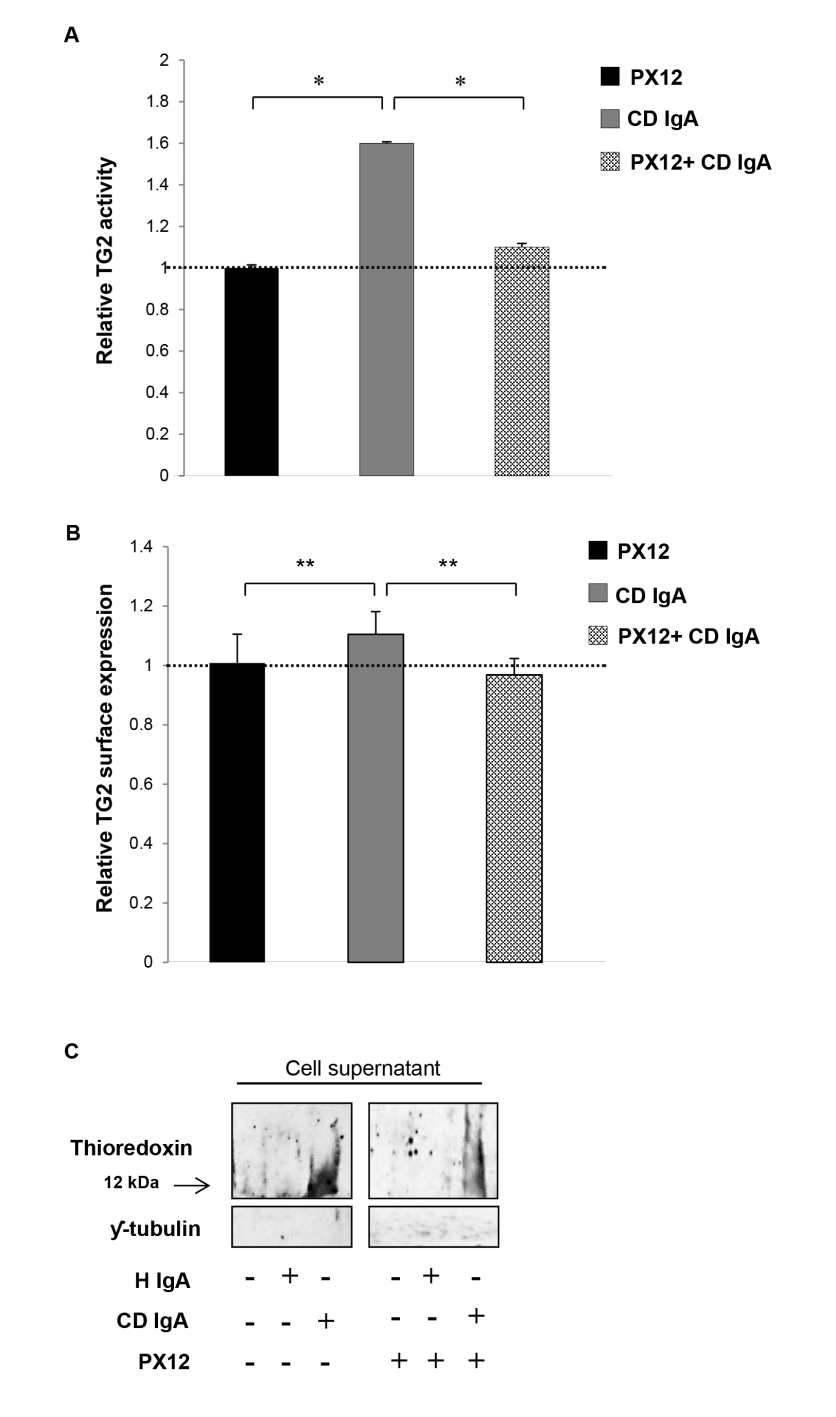
Celiac IgA CD IgA) mediated activation of extracellular transglutaminase 2 (TG2) by thioredoxin (TRX). (**A**) The relative surface expression of TG2 as well as (**B**) the relative TG2 activity on HUVECs treated with CD IgA autoantibodies was investigated by ELISA prior preincubation of endothelial cells with TRX inhibitor, PX12. The dashed line indicates TG2 expression and activity in untreated HUVECs. Bars represent mean values as fold of control and error bars indicate standard error of the mean. P-value < 0.05 was considered significant ( _*_p<0.01 and _**_p<0.001). (**C**) The secretion of TRX in endothelial cell (HUVECs) culture media was analysed by Western blotting in the presence of a specific TRX inhibitor, PX12 (1-methylpropyl 2-imidazolyl disulfide). Representative Western blots of HUVECs culture supernatants show amounts of TRX and ƴ-tubulin used as loading control for quality sample. Data derived from at least three independent experiments, repeated in quadruplicate are shown. Endothelial cells were treated with CD IgA and non-celiac subject’s immunoglobulin-A (H IgA).

In view of these observations, we next sought to establish whether inhibition of extracellular TRX by PX12 might counteract the effects of CD IgA autoantibodies observed on HUVECs. Such seemed indeed to be the case, as pretreatment of endothelial cultures with PX12 prevented the extracellular TG2 activation mediated by CD IgA (Figure 6A). In addition to this, inhibition of extracellular TRX with PX12 was able to overcome the effects mediated by CD IgA on TG2 surface expression and secretion of TRX into the culture supernatants (Figure 6B and 6C).

## Discussion

Our previous early studies indicated that CD IgA disturbs angiogenesis, at least *in vitro*, presumably by increasing extracellular TG2 transamidase activity, but the underlying mechanism here is poorly understood [15,16]. Here we show that the administration of CD IgA to confluent HUVEC monolayers starved O/N, leads to an early and constitutive activation of extracellular TG2 (Figures 1A and 2A). Interestingly, we observed no time dependent correlation with the transamidating activation of TG2 by CD IgA, as the induction was detectable at all-time points examined (Figures 1A and 2A). We did, however, note a marked CD IgA dose-dependent activation of extracellular TG2 (Figure 1B), in line with previous work reporting dose-dependent enhancement of TG2 transamidase activity by celiac autoantibodies [22]. The correlation between the amount of disease-specific autoantibodies and TG2 enzymatic activity has also been documented in other previous articles [23,24], but with contrasting outcomes as regards the activation of the protein. In fact, both groups show that disease specific autoantibodies had an inhibitory effect on TG2 enzymatic activity, this possibly due to different experimental conditions [23,24].

In view of evidence which correlates increased extracellular TG2 enzymatic activity with high TG2 expression levels [19,25-27] we sought to investigate whether CD IgA-increased extracellular TG2 transamidating activity was associated with augmented cell surface TG2 presence. We have previously shown that celiac disease autoantibodies do not up-regulate the total TG2 protein expression level in endothelial cell lysates [15]. In contrast, in endothelial cells treated with CD IgA autoantibodies there was a small but statistically significant increase in the relative TG2 presence on the cell surface (Figure 3A and 3B) which nonetheless did not account for the increased extracellular TG2 activity. This supports the hypothesis that extracellular TG2 activation by CD IgA in endothelial cell is a more complex and elaborate process involving other important biological players. If the increased extracellular TG2 expression in the presence of CD IgA has a physiological significance or not, needs to be further investigated.

Bearing in mind that TG2 is subject to oxidation and as a result gradually loses activity upon handling and storage [20], we examined how the reducing or oxidizing environmental conditions might affect CD IgA-mediated modulation of TG2. Interestingly, in an *in vitro* system, CD IgA was able of inducing TG2 activation only in the presence of a reducing agent without the addition of exogenous Ca^2+^ (Figure 4A), supporting the conception that most probably their biological effects in endothelial cells are exerted under a reducing extracellular environment. Moreover, here we have shown that oxidation alters the protein’s conformation in such ways that the antigenic epitopes are less accessible and thus the reducing environment helps maintain longer and more effective binding of the antibodies to the TG2 antigen (Figure 4B). Recently, Jin and co-workers [14] elegantly demonstrated in cell culture that extracellular TG2 can be activated by the cellular secreted redox protein TRX, which helps to keep the protein in an open and active conformation. Interestingly, we found here that CD IgA-treated HUVECs secreted TRX into the extracellular space and that this was coupled with reduced surface expression of the protein (Figure 5A and 5B), this suggesting that TRX might have a role in CD IgA-mediated TG2 activation. Such assumption was supported by our results in that pretreatment of endothelial cultures with PX12 prevented the extracellular TG2 activation mediated by CD IgA (Figure 6A). It is of note that, PX 12, similarly to other alkylating agents [28], was able to prevent TG2 enzymatic activity in the presence of Ca^2+^ and DTT. Further to the above, we observed that the inhibition of extracellular TRX by PX12 was able to prevent secretion of the protein and to normalize TG2 surface expression in endothelial cells treated with CD IgA but did not prevent its decrease from the surface (data not shown). Previous works have shown that the pro-inflammatory cytokine INF-γ can induce secretion of TRX at levels capable of activating TG2 [29]. In contrast, our findings exclude a role for INF-γ in the constitutive activation of extracellular TG2 mediated by CD IgA, since this was not detectable in our experimental settings (data not shown). In summary, our data suggest that CD IgA mediates TG2 activation in the extracellular environment where the protein is mainly inactive under physiological conditions [30], by a mechanism involving the redox sensor protein TRX. In our system, disease-specific autoantibody-induced INF-γ-independent secretion of TRX probably helps to keep TG2 in a conformation suitable for the constitutive antibody binding and activation described in the present work. Intriguingly, the decreased surface expression of TRX and the augmented TG2 expression on HUVECs may lead us to speculate that at the cell surface TG2 might be present in a conformation which is not suitable for the enzymatic activation mediated by CD IgA.
